# Clinical Methods

**Published:** 1917-04

**Authors:** 


**Clinical Methods.** By ROBERT HUTCHISON, M.D., F.R.C.P., and HARRY RAINY, M.D., F.R.C.P., F.R.S.E. Pp. viii. 654. London: Cassell & Co. 1916. Price 10s. 6d.

The sixth edition of this well-known guide to the practical study of medicine has been revised throughout. The most important changes are in the chapter on Clinical Bacteriology, which has been largely re-written by Professor James Ritchie. Several of the newer organisms, which have derived recent importance from their connection with the war, are dealt with; amongst them are the Bacillus dysenteriæ, B. tetani, Micrococcus rheumaticus, and the Diplococcus intracellularis of cerebro-spinal fever. The book has become increasingly useful to students who are commencing their clinical work, and it will be years before they have assimilated the mass of information it contains.

